# Epigenetics in depression and gut-brain axis: A molecular crosstalk

**DOI:** 10.3389/fnagi.2022.1048333

**Published:** 2022-12-13

**Authors:** Nusrat Begum, Aniket Mandhare, Kamatham Pushpa Tryphena, Saurabh Srivastava, Mohd Farooq Shaikh, Shashi Bala Singh, Dharmendra Kumar Khatri

**Affiliations:** ^1^Cellular and Molecular Neuroscience Laboratory, Department of Pharmacology and Toxicology, National Institute of Pharmaceutical Education and Research, Hyderabad, Telangana, India; ^2^Department of Pharmaceutics, National Institute of Pharmaceutical Education and Research, Hyderabad, Telangana, India; ^3^Neuropharmacology Research Strength, Jeffrey Cheah School of Medicine and Health Sciences, Monash University Malaysia, Bandar Sunway, Selangor, Malaysia

**Keywords:** gut-brain axis (GBA), microbiota, epigenetic, depression, microbiome, short-chain fatty acid (SCFA), DNA methylation, histone modifications

## Abstract

Gut-brain axis is a dynamic, complex, and bidirectional communication network between the gut and brain. Changes in the microbiota-gut-brain axis are responsible for developing various metabolic, neurodegenerative, and neuropsychiatric disorders. According to clinical and preclinical findings, the gut microbiota is a significant regulator of the gut-brain axis. In addition to interacting with intestinal cells and the enteric nervous system, it has been discovered that microbes in the gut can modify the central nervous system through metabolic and neuroendocrine pathways. The metabolites of the gut microbiome can modulate a number of diseases by inducing epigenetic alteration through DNA methylation, histone modification, and non-coding RNA-associated gene silencing. Short-chain fatty acids, especially butyrate, are well-known histone deacetylases inhibitors. Similarly, other microbial metabolites such as folate, choline, and trimethylamine-*N*-oxide also regulate epigenetics mechanisms. Furthermore, various studies have revealed the potential role of microbiome dysbiosis and epigenetics in the pathophysiology of depression. Hence, in this review, we have highlighted the role of gut dysbiosis in epigenetic regulation, causal interaction between host epigenetic modification and the gut microbiome in depression and suggest microbiome and epigenome as a possible target for diagnosis, prevention, and treatment of depression.

## Introduction

Microbiota study has advanced dramatically over the past 20 years, and it is now becoming more evident how these microscopic inhabitants affect our daily lives in many ways. The microbiota plays a significant role in determining human health and disease and controlling host physiology. Several international projects such as Human Microbiome Project, Metagenomics of the Human Intestinal Tract, International Human Microbiome Consortium have characterized the human microbiota (Bäckhed et al., 2012; Huttenhower et al., 2012) which includes various microbiome such as gut microbiome, oral microbiome, vaginal microbiome, skin and placental microbiome. The gut microbiota has abundance of two bacterial phyla, the gram negative *Bacteroidetes* and the gram positive *Firmicutes*; *Actinobacteria, Fusobacteria*, and *Verrucomicrobia* levels are comparatively lower and varies remarkably among individuals (Arumugam et al., 2011; Bäckhed et al., 2012). The vaginal microbiome consists of over 200 phyla of which *Firmicutes, Bacteroides*, *Actinobacteria* and *Fusobacteria* are the predominant phyla (Romero et al., 2014). *Lactobacillus* sp. plays a major role in maintaining the acidic pH of vagina by secretion of lactic acid and hydrogen peroxide, failure to do so results in bacterial vaginosis, an ecological disorder of vaginal microbiota (Kenyon et al., 2013). The oral microbiome comprises of bacteria, fungi, viruses, protozoa, and archaea. It includes more than 20 bacterial phyla spread over more than 300 genera (Zhou et al., 2013; Jiang et al., 2014). Skin microbiome comprises more than 100 microbial phylotypes, both harmless or beneficial for host, which differ in their abundance and diversity depending upon race, skin color, and geographic location (Rosenthal et al., 2011; Ladizinski et al., 2014). The placental microbiome is composed of non-pathogenic commensal bacteria derived from phylum *Firmicutes, Proteobacteria*, *Bacteroidetes*, and *Fusobacteria* (Aagaard et al., 2014). The population of placental microbiota differ in preterm (gram negative *Burkholderia*) and normal delivery (gram positive *Paenibacillus*) (Groer et al., 2014).

**FIGURE 1:**
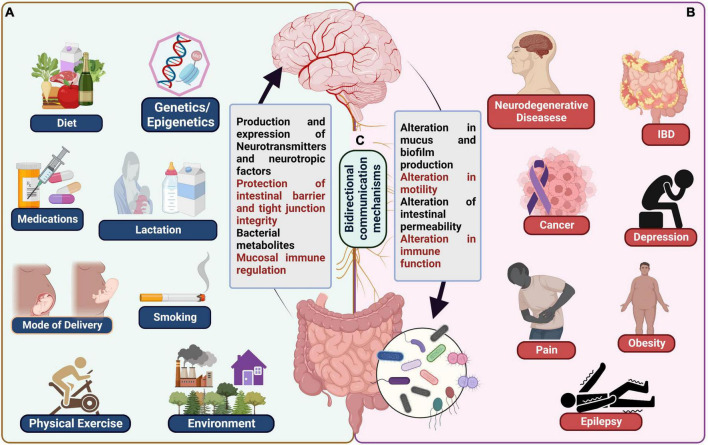
**(A)** Examples of typical influences on the microbiota gut-brain axis including genetics/epigenetics, medications, mode of delivery, lactation, smoking, physical exercise, and environment. **(B)** Various diseases known to be affected by gut brain axis dysbiosis, including neurodegenerative diseases, cancer, IBD, depression, obesity, pain, and epilepsy. **(C)** Bidirectional communication mechanisms between the gut and the brain.

Various environmental factors influence the composition of gut microbiota. They include macro environmental factors like socio economic, chemical, built environment and micro environmental factors like smoking, alcohol consumption, dietary habits (Ahn and Hayes, 2021). Many studies provide evidence that chemical substances like arsenic, triclocarban, triclosan upon being metabolized by gut microbiota in turn cause gut dysbiosis (Rook et al., 2014; Halden, 2016). The association between gut microbiota and built environment (infrastructures built by humans) is an emerging hypothesis and yet to be explored further (Ahn and Hayes, 2021). Low socioeconomic status, psychosocial stress, sedentary lifestyle also influences the gut microbiota as evidenced in studies conducted in some countries (Lin et al., 2013). Similarly, studies found that smokers had decreased diversity of gut microbiome. The studies demonstrate that Phyla *Proteobacteria, Bacteroidetes* and genera *Clostridium, Bacteroides, Prevotella* were increased and *Actinobacteria, Firmicutes* phyla were decreased (Savin et al., 2018). Chronic Alcohol consumption was found to alter the gut microbiota resulting in decreased *Bacteroidetes* and increased *Proteobacteria* (Mutlu et al., 2012). Finally, dietary habits like consumption of high fat, sugar, protein, and fiber intake also affect the gut microbiota composition. High fiber diet was proved to have beneficial effect on gut microbiome by speeding up the proliferation of microbiota as well as increase the diversity (Donohoe et al., 2014). A study conducted in Africa showed that children exposed to antibiotics and indoor cooking fires had decreased diversity and dysbiosis of gut microbiota (Nel Van Zyl et al., 2021). Food additives, organic pesticides, heavy metals also cause gut dysbiosis (Jin et al., 2017). Recent studies have emphasized the role of the microbiota in average intestinal growth and function, including digestion and nutrition intake, metabolism, tissue formation, and immunity (Hooper et al., 2012). Furthermore, alterations in the microbiota’s composition or abundance have been linked to several chronic human illnesses, including local diseases like inflammatory bowel disease (IBD) (Nagao-Kitamoto et al., 2016), metabolic diseases like obesity and diabetes (Napolitano and Covasa, 2020), cancer (Vivarelli et al., 2019) and neuropsychiatric disorders like autism (Iglesias–vázquez et al., 2020), schizophrenia (Szeligowski et al., 2020) or depression (Liu et al., 2021; Figure 1).

Depression ranks second in the global disease burden ranking (Smith, 2014). Its heritability is only 37% (Sullivan et al., 2000) which is far less than other psychiatric diseases, e.g., Schizophrenia and bipolar disorders which account for 70–80% (Kendler, 1983). Previous research revealed an approximately 50% variability rate of depression in monozygotic twins, which indicates the involvement of various other non-genetic factors in the pathogenesis of depression (Fraga et al., 2005). Identification of gene loci using human genome-wide association studies failed to give reproducible results (Bosker et al., 2011). The factors involved in resilience and susceptibility to depression include variability penetrant, genetic differences, and environmental factors (Sun et al., 2013). Environmental factors such as lifestyle, diet, physical exercise, and stress can affect gut microbiome composition and epigenetic alterations. Various studies have provided insight into how the metabolites produced by the gut microbiome act as an epigenetic regulator by modifying epigenetic mechanisms such as DNA methylation (Kovacheva et al., 2007; Kok et al., 2015), histone modifications (Soliman et al., 2011; Krautkramer et al., 2016; Wang et al., 2019), or non-coding RNA associated silencing (Liang et al., 2015; Paul et al., 2015; Virtue et al., 2019). Despite the tremendous research, there are still significant gaps in our knowledge of how epigenetic mechanisms contribute to depression. Understanding of the pathological mechanisms of depression will advance with the closure of knowledge gaps on the multifactorial interaction between epigenetics, gut microbiome, and their antidepressant effects, which may also help in the development of more sophisticated pharmacological approaches. Thus, in this review, we have tried to establish a connection between gut dysbiosis, and epigenetics to suggest them as a possible target for diagnosis and treatment of depression.

## Gut- brain axis

The gut-brain axis (GBA) is a complex bidirectional network between gut microbiota and the brain (Iannone et al., 2019). It consists of several immunological, endocrinological, and neural mediators. Gut microbiota is a collection of all microbial strains present in a gut. Microbiota development is host-specific and develops fully in the first 3 years of life. Microbiota composition varies from individual to individual and is generally based on environmental factors, mode of delivery, food intake, and disease condition (Burokas et al., 2015). Intestinal microbiota, central nervous system (CNS), and enteric nervous system (ENS) mediate the GBA (Ambrosini et al., 2019). More than 75% of gut microbiota comprises *Bacteroidetes* and *Firmicutes* species. Depending upon the bacterial strain in the intestine, this microbiota can regulate various brain functions, such as alteration of memory, increased stress response, and anxiety-like behavior (Evrensel et al., 2020). The composition of gut microbiota differs from individual to individual. Changes in the balance of common gut microbiota affect the production of fermentation products of bacteria such as short-chain fatty acids (SCFAs), acetic acid, propionic acid, and butyric acid, which play an essential role in CNS function and regulate intestinal adaptive response (de Vadder et al., 2014).

The effect of microbiota on physiology and disease can be clearly understood by utilizing germ-free (free of all microorganisms; obtained via c-section and raised in sterile isolators) and Specific Pathogen Free (SPF) (free of specific disease-causing pathogens capable of altering mouse health and research outcome) mice. Experimental studies in Germ-free (GF) mice showed that gut microbiota is required to develop the hypothalamus pituitary adrenal (HPA) axis. GF mice showed increased levels of adrenocorticotropic hormone (ACTH) and corticosterone when exposed to stress conditions compared to normal and SPF mice. Studies also showed decreased BDNF expression levels in the cortical and hypothalamic regions of the brain in GF mice compared to SPF mice (Sudo et al., 2004).

## Composition of gut microbiota

The gastrointestinal tract of human beings contains 100 trillion different types of micro-organisms, which include bacteria, viruses, and yeast (Shi et al., 2017; Rinninella et al., 2019). Nearly 1,000–1,500 bacterial species are included in the gut microbiota, but only 160 species are present in any individual (Shi et al., 2017; Rowland et al., 2018). This shows that the microbiota between two individuals is different (Shi et al., 2017). The dominant phyla of bacteria in the gut are *Actinobacteria, Bacteroidetes, Firmicutes, Fusobacteria, Proteobacteria*, and *Verrucomicrobia. Firmicutes* and *Bacteroidetes* constitute 90% of the gut microbiota. *Lactobacillus, Bacillus, Clostridium, Enterococcus*, and *Ruminococcus* constitute 95% of the *Firmicutes*, whereas *Bacteroides* and *Prevotella* are dominant among *Bacteroidetes* (Rinninella et al., 2019). Initially it was thought that the colonization of microbiota takes place during delivery and after birth. But with the advent of several molecular techniques like fluorescent *in situ* hybridization, Polymerase chain reaction etc., it has been revealed that the fetus encounters the commensal as well as pathogenic bacteria through umbilical cord blood, amniotic fluid, placenta, and fetal membrane (Stinson et al., 2016). It has been demonstrated that this encounter is necessary for the development of gastrointestinal tract in the fetus through the use of GF animal models (Chowdhury et al., 2007; Le Hurou-Luron et al., 2010). In some studies, bacteria of the adult gastrointestinal tract like *E. coli, Enterococcus* sp. had been found in the placenta, meconium and amniotic fluid respectively (DiGiulio et al., 2008; Aagaard et al., 2014). Proteobacteria were predominantly found in the amniotic fluid (Doyle et al., 2017). Duration of pregnancy also effects the composition of the neonates gut microbiota. *Clostridium* sp., *E. coli, Enterococcus, Streptococcus, Staphylococcus, Klebsiella* were predominantly found in the gut of premature infants. Maternal health, dietary habits, antibiotic intake etc influence the fetal gut microbiota composition (Hill et al., 2017). But results from some other studies prove the opposite (Kim et al., 2009; Boro et al., 2014). Hence it is still doubtful to conclude about the prenatal colonization and composition of gut microbiota. More studies with appropriate experimental design coupled with advanced analyzing techniques in the future might provide clear insights about this controversy. According to Gosalbes et al. (2013), two types of meconium microbiota are present, a less diverse *Enterobacteriaceae* predominant microbiota and a highly diverse *Firmicutes* predominant microbiota (Tanaka and Nakayama, 2017). Extensive colonization starts at birth during the delivery and breastfeeding. Gestational age, mode of delivery, feeding method, sanitation, and antibiotic treatment are some factors that influence the colonization of the infant’s gut. The diversity in the gut microbiota of neonates is low. *Proteobacteria* and *Actinobacteria* are the predominant phyla in the gut microbiota of neonates. Initially, the facultative anaerobes colonized and pave the way for the colonization of strict anaerobes. Mother’s milk is another primary source of microbiota which includes *streptococci* and *staphylococci* (Rodríguez et al., 2015). The breastfed infant’s gut microbiota consists of a more significant number of *Bifidobacterium* species that live on the Human Milk Oligosaccharides (HMO). The diversity of the gut increases after weaning and commencement of solid food (Rodríguez et al., 2015; Milani et al., 2017). *Bacteroidetes* and *Firmicutes* (*Clostridia*) help break down complex carbohydrates that predominate the gut microbiota after the introduction of solid food (Derrien et al., 2019). The child’s oral bacteria also colonize the gut (Rodríguez et al., 2015). The diversity of microbiota progresses with age (Rodríguez et al., 2015; Rinninella et al., 2019). Initially, it was thought that, as the child reaches age three, the composition and diversity of the gut microbiota becomes identical to that of the adult (Cresci and Bawden, 2015; Rinninella et al., 2019). But many studies suggest that gut microbiota composition continues to develop even after 3 years of age (Derrien et al., 2019). *Bifidobacterium* is present at higher levels in children than in adults (Derrien et al., 2019). It remains almost constant until the person ages (Cresci and Bawden, 2015). *Firmicutes, Bacteroidetes*, and *Actinobacteria* dominate the adult gut microbiota. As age progresses, the microbiota composition is altered due to a decrease in dentition, reduced salivary function and digestion, and alteration in intestinal transit time (Cresci and Bawden, 2015). After 70 years of age, it has been observed that anaerobic bacteria like *Bifidobacterium* decreases whereas *Proteobacteria* and *Clostridium* increase in abundance (Rinninella et al., 2019). Other than bacteria, methanogens of the archeal order, like *Methanobrevibacter smithii* and *Methanosphaera stadtmanae*, are also present in the adult gut microbiota (Derrien et al., 2019). Fungal cells are also present in gut microbiota but in less number than the bacteria. Fungi, like *Aspergillus* species, tremellomycetes are more predominant in children than in adults (Derrien et al., 2019).

The gut microbiota composition varies between individuals and depends on the factors like enterotypes, body mass index, lifestyle, ethnicity, dietary and cultural habits, exercise frequency (Rinninella et al., 2019), genetics (Wu et al., 2011; Rodríguez et al., 2015; Shi et al., 2017), stress, antibiotic use (Cresci and Bawden, 2015), geography (Derrien et al., 2019). Enterotypes are the specific clusters of bacteria that characterize the individual’s gut microbiota. There are three enterotypes: enterotype I, in which *Bacteroides* is the dominant cluster, *Prevotella* in enterotype II, and *Ruminococcus* in enterotype III. Each enterotype generates energy from the fermentable substrates present in the colon in a specific way (Rinninella et al., 2019) and is closely linked to long-term dietary patterns (Wu et al., 2011). Consumption of a high-fiber diet is associated with increased microbial diversity and stability (Derrien et al., 2019).

## Metabolites of gut microbiota

There is a mutually beneficial symbiotic relationship between the host and the gut microbiota (Shi et al., 2017). The microbiome, which is the collection of the microbiota genomes, harbors different genes that encode different types of proteins that are not encoded by the human genome (Kho and Lal, 2018). Gut bacteria play an essential role in the digestion of food, absorption of nutrients, and production of metabolites like SCFAs, lipids, vitamins, bile acids, branched-chain amino acids, tryptophan, and indole derivatives (Rinninella et al., 2019; Agus et al., 2021;Table 1). Gut microbiota also maintains the integrity of the intestinal epithelium and prevents bacterial invasion and pathogenic colonization in the gut (Rinninella et al., 2019).

**TABLE 1:** Chief metabolites produced by the gut microbiota.

Chief metabolites produced by gut microbiota	Important functions	References
SCFAs Ex: Acetate, propionate, butyrate	Maintain the integrity of intestinal mucosa, regulate lipid and glucose metabolism, regulation of the immune system, control inflammatory responses, regulates appetite, maturation of microglia, play a role in the synthesis and release of neurotransmitters like 5-HT, acetylcholine from ECC, nor-adrenaline from sympathetic neurons	Koh et al., 2016; Morrison and Preston, 2016; Kho and Lal, 2018; Caspani et al., 2019; Agus et al., 2021
Branched chain amino acids Ex: Valine, isoleucine, leucine	Regulate the synthesis of proteins, metabolism of glucose and lipids, immunity, proliferation of Hepatocyte, insulin resistance	Koh et al., 2016
Secondary bile acids Ex: Deoxycholic acid, lithocholic acid	Metabolic functions like promoting glycogen synthesis, secretion of insulin, thermogenesis, mediation of satiety, anti-inflammatory properties, anti-microbial effects	Koh et al., 2016; Kho and Lal, 2018; Rowland et al., 2018
Tryptophan and indole derivate metabolites Serotonin, aryl hydrocarbon receptor agonists like indole-3-propionic acid (IPA)	Regulation of tryptophan metabolism in the cells, induce the production of serotonin (5-HT3), IPA has neuroprotective action. It inhibits the amyloid beta fibril formation	Koh et al., 2016; Kho and Lal, 2018
Vitamins Vitamin K and B group	Vitamin K2 reduces the risk of cardiovascular disease Vitamin B5, B12 are necessary for neurologic functioning Red blood cell production Cofactors for various biochemical reactions	Kho and Lal, 2018
Polyphenol metabolites Ex: Urolithin A&B, equol, *n*-phenyl naringenin	Reduces oxidative stress, estrogen modulation, platelet inhibition, anti-inflammatory, and anti-microbial effects	Kho and Lal, 2018; Rowland et al., 2018
Neurotransmitters Ex: GABA, 5-HT, acetylcholine, dopamine	Modulate the neurotransmission by acting on the CNS receptors or receptors present on the peripheral neurons or immune cells, afferent nerves or VN	Caspani et al., 2019

### Short chain fatty acid

Short chain fatty acid are produce in colon by gut microbiota as carbon and energy source through fermentation of complex and indigestible carbohydrates (Hugenholtz et al., 2013). Upon microbial hydrolysis of carbohydrate, pyruvate (a key precursor for SCFA) is produced by glycolytic pathway for hexoses and pentoses (Hugenholtz et al., 2013). The pathway for butyrate and propionate production are substrate specific and more conserved whereas pathways for acetate production are commonly spread among various bacterial classes (Morrison and Preston, 2016). *Bacteroidetes* and *Negativicutes* uses succinate pathway whereas *Lachnospiraceae* uses propanediol pathway for propionate production (Louis and Flint, 2017). The butyrate production is mediated by enzymes butyrate kinase (*Coprococcus comes* and *Coprococcus eutactus*) and butyryl coA-acetate coA transferases (*Faecalibacterium prausnitzii, Eubacterium rectale*, and *Eubacterium hallii*) (Venegas et al., 2019). The acetate, butyrate and propionate contribute 60, 20, and 20% respectively in total SCFA (Cummings et al., 1987). SCFAs are essential in maintaining the intestinal epithelium’s integrity by regulating the tight junction proteins. They also preserve glucose homeostasis through Free Fatty Acid Receptors 2/3 (FFAR2/3). Butyrate and acetate are lipogenic, whereas propionate is gluconeogenic in nature. They also play a role in gut-hormone-derived signals. Reduction in lipolysis and adipogenesis is seen with increased SCFAs in circulation. They also play an essential role in regulating appetite by modulating neuronal activity and visceral reflexes. SCFAs, especially butyrate, play a vital role in the regulation of the immune system and inflammation by inhibiting the Nuclear Factor Kappa B (NF-kB) activation in macrophages and also deter Histone Deacetylases (HDAC) (Koh et al., 2016; Morrison and Preston, 2016). SCFAs also regulate the microglia’s maturation and function in the CNS (Koh et al., 2016; Kho and Lal, 2018).

### Vitamins

The gut microbiota has a vital role in synthesizing vitamins. They synthesize vitK2, which reduces the risk of cardiovascular diseases. They also produce vitamins B5, and B12, which are essential for proper neurological functioning (Kho and Lal, 2018). The gut microbiota also synthesizes group B vitamins, thiamine, riboflavin, nicotinic acid, pantothenic acid, pyridoxine, biotin, folates, and cyanocobalamin (Rowland et al., 2018). Vitamin B5 and B12 are necessary for proper neurological functioning (Kho and Lal, 2018).

### Secondary bile acids

The gut microbiota also plays a vital role in the metabolism of bile salts by secreting bile salt hydrolases and converting unabsorbed primary bile salts to secondary bile salts and deconjugating them. Then they are partly reabsorbed, and the remaining are excreted by the host. These secondary bile acids act as ligands for the host’s Farsenoid X nuclear receptors (FXR) and have anti-microbial effects (Kho and Lal, 2018; Rowland et al., 2018).

### Polyphenols

The gut microbiota also metabolizes polyphenols that are present in fruits and vegetables into simpler forms that are easily absorbed (Rowland et al., 2018). Tryptophan derivatives and indole derivatives, are also produced by gut microbiota (Koh et al., 2016; Kho and Lal, 2018; Caspani et al., 2019). Gut microbiota also plays an essential role in the absorption of minerals like iodine, iron, zinc, selenium, and copper (Bargiel et al., 2021).

Some metabolites like trimethyl-*N*-oxide (TMO), a metabolite produced from choline-containing foods are associated with cardiovascular risk. Similarly, Imidazole Propionate, a histidine utilization product, is found to increase insulin resistance and is associated with type 2 diabetes (Agus et al., 2021).

### Neurotransmitters

The most obvious method by which the microbiota could affect the neurological system is by altering host neurotransmitters and/or associated pathways. In fact, it has been discovered that a variety of important neurotransmitters can be produced by gut microbiota. It’s interesting to note that some bacterial species in the gut can also produce enzymes to facilitate the synthesis of neurotransmitters or their precursors. Additionally, some gut bacteria can communicate with intestinal enteroendocrine cells (EEC) via their metabolites to control the production and release of neurotransmitters (Yano et al., 2015; Kaelberer et al., 2018, 2020). It was recently discovered that a subpopulation of intestinal EEC synthesizes glutamate and uses it to send quick signals to the brain through the vagus nerve (VN) (Kaelberer et al., 2018). “Neuropod cells” are EEC that synapses with vagal neurons; they express the vesicular glutamate transporter 1 (VGLUT1) gene and release glutamate to transmit sensory stimuli from gut sugars to the brain in milliseconds (Frost et al., 2014). Coculture technique used in a recent study to find the key growth factor for bacterial survival revealed that gram positive human gut bacteria from *Ruminococcaceae* family require *Bacteroides fragilis* for their growth (Strandwitz et al., 2019). It was later found that *B. fragilis* primarily produces GABA as a growth factor. Additionally, this co-culture system demonstrated that *Bifidobacterium, Parabacteroides*, and *Eubacterium*, also synthesize GABA. Since it cannot cross the BBB, GABA produced by gut microbes may instead act locally on the VN or ENS. The early 1900s saw the discovery of acetylcholine (Ach) in a study of ergot on wheat rye; however, it wasn’t until much later that it was realized that *Bacillus acetylcholini*, not ergot, was the true producer of this neurochemical (O’Donnell et al., 2020). Since then, numerous microbes, including *B. subtilis*, *L. plantarum*, *E. coli*, and *S. aureus*, have been discovered to produce Ach. The Ach levels are notably higher in *B. subtilis* than *E. coli* or *S. aureus* (Koussoulas et al., 2018). *Staphylococcus* has been found to produce dopamine in the human intestine by the enzyme staphylococcal aromatic amino acid decarboxylase (SadA) which allows it to take up the precursor L-3,4-dihydroxy-phenylalanine (L-DOPA) and convert it into dopamine (Luqman et al., 2018). In humans, more than 50% of dopamine is synthesize in gut (Eisenhofer et al., 1997). Mucosal blood flow, gastric secretion and motility are regulated by dopamine and its receptor in the GIT (Vaughan et al., 2000; Al-Jahmany et al., 2004). In the human body, enterochromaffin cells (ECC), primarily in the intestinal epithelium, synthesize over 90% of serotonin. The bacterial kynurenine production pathway regulates how ECC in the gut use the amino acid tryptophan from dietary protein as a substrate to produce serotonin (Bailey and Cryan, 2017). Spore-forming bacteria in the gut (mostly *Clostridia*) might encourage the biosynthesis of serotonin by raising the gene expression of the rate-limiting enzyme tryptophan hydroxylase 1 (TPH1) in colonic ECC (Yano et al., 2015). *Staphylococci* have also been found to produce serotonin by decarboxylating the precursor 5-hydroxytryptophan (5-HTP) into serotonin using the enzyme SadA (Luqman et al., 2018). Numerous studies have shown that, in the absence of microbial colonization (in GF mice), level of neurotransmitters (GABA, serotonin, and Ach) as well as their precursors (tryptophan and choline) changes in the feces and serum (Yano et al., 2015). Similarly, acquired deprivation of gut microbiota (through antibiotic treatment) also results in changes in level of neurotransmitter and their precursors in gut and blood (Gao et al., 2019). It’s interesting to note that variations in gut microbial diversity also affect the expression of neurotransmitter receptors in the brain (Sudo et al., 2004; Bravo et al., 2011; Neufeld et al., 2011).

## Factors affecting modulation of gut-brain axis

### Prebiotics and probiotics

Prebiotics are fermentable ingredients utilized by bacteria and modulate bacterial activity by promoting the growth of symbiotic bacteria and decreasing the growth of pathogenic bacteria. In comparison, probiotics are living strains of microorganisms. Generally, prebiotics are beneficial and consumed by microbial flora, and these prebiotics are found in onion, wheat, and leafy vegetables in the form of polyphenols or oligosaccharides. A prebiotics-rich diet such as dietary fibers, oligosaccharides, and inulin significantly alters the microbial composition and activity. Bimuno-galactooligosaccharide consumption as a prebiotic therapy showed a decrease in the salivary cortisol awakening response and an improvement of mood and behavior in healthy volunteers compared to placebo (Schmidt et al., 2015).

Numerous advantages substantiated by research have been associated with regular consumption of probiotic foods and supplements includes reduction in inflammation (Hilton et al., 1997; Lai et al., 2019), management of diarrhea and other digestive complications (Hilton et al., 1997; Salazar-Lindo et al., 2004; Lahtinen et al., 2011; Aggarwal et al., 2014; Eskesen et al., 2015; Lai et al., 2019) as well as wide spectrum of other conditions from autoimmune diseases (Pham et al., 2008; Takada et al., 2017; Parker et al., 2018; Nishida et al., 2019; Ansari et al., 2020; Chao et al., 2020), emotional imbalance to cancer (Liu et al., 2011; Bajramagic et al., 2019; Sasidharan et al., 2019). Lactic acid bacteria, namely *Bifidobacterium* and *Lactobacillus* strains, are the most prevalent bacterial species used in modern probiotic products (Spano et al., 2019). Other bacterial species, such as *Faecalibacterium prausnitzii* and *Akkermansia muciniphila*, have been found in recent studies to possibly have positive effects when used as probiotics (Kumari et al., 2021). Their use in probiotic products is increasing as well (Saarela, 2019). The oral supplementation of *B. fragilis* has improved the gut permeability and microbial composition in maternal immune activation mouse model of autism spectrum disorder (Hsiao et al., 2013). Similarly, improvement in memory and learning was observed in rat model of AD upon supplementation of probiotics (*L. acidophilus, L. fermentum, B. lactis*, and *B. longum*) in drinking water for 8 weeks (Azm et al., 2018). Probiotics cannot always be considered an alternative to medication, especially in situations of severe disorders, despite the fact that many of their clinical advantages have undergone thorough testing. Early maternal separation causes a decrease in the expression of Corticotropin-releasing hormone receptor 1 (CRH R1) in the hippocampal region due to dysregulation of the HPA axis (Nemoto et al., 2015). Probiotic therapy of *Lactobacillus rhamnosus* GG in rodent studies showed a significant increase in the expression of CRH R1 in the hippocampal region and restoration of HPA activity (McVey Neufeld et al., 2019).

### Birth

Mother-to-infant transmission is the first and primary approach to inhabiting gut microbiota during birth. Fecal examination of vaginally delivered infants showed a significant increase in the population of *Bifidobacterium* species such as *Bifidobacterium adolescentis, B. catenulatum*, and *B. longum* when compared with cesarean-born infants, which means the mother’s intestine and vaginal delivery are one of the crucial factors which regulate growth and species present in gut microbiota (Makino et al., 2013). As the infant grows, a sufficient number of prebiotics are given through breastfeeding as it is required for normal growth of microbial flora and different strains of *lactobacillus* species (Haarman and Knol, 2006).

### Diet

Dietary intake and lifestyle greatly influence gut microbiota composition. Numerous popular diets, such as the Western (Drasar et al., 1973; Reddy et al., 1975; Wu et al., 2011), Mediterranean (Lopez-Legarrea et al., 2014; De Filippis et al., 2016), vegetarian, vegan, and gluten-free diets (Sanz, 2010; Pisarello et al., 2015; Bonder et al., 2016), have been investigated for their capacity to modify gut microbiota. The western diet (high protein/animal fat) is proved to increase *Bacteroides* and *Enterobacteria* while reducing the diversity of *Eubacterium* and *Bifidobacterium* sp. The Mediterranean diet (high fiber and antioxidants, low in red meat, high monounsaturated fat) is believed to reduce inflammation, improve lipid profiles, and reduce the risk of obesity (Sandhu et al., 2017). In terms of microbes, these traits were linked to increase in *Prevotella, Bifidobacterium*, and *Lactobacillus*, and decrease in *Clostridium* (Clemente-Postigo et al., 2012; Queipo-Ortuño et al., 2012; Fava et al., 2013; Koloverou et al., 2016). When compared to an unrestricted control diet, vegan and vegetarian diets were found to dramatically decrease the levels of *Bacteroides* and *Bifidobacterium* sp. (Wu et al., 2016). Incompetent dietary consumption reduces microbial nutrient intake and decreases the population of beneficial microbial flora. As a compensatory mechanism, microbial flora starts consuming glycoprotein-rich protective mucosal barrier, which can further lead to the leaky wall and dysbiotic condition to initiate inflammation (Tabrizi et al., 2019). Studies found that an inulin or Oligofructose-rich diet is responsible for improving memory, mood and cognition enhancement (Smith et al., 2015).

### Obesity

The difference in metabolic phenotype was observed between lean and genetically obese (fa/fa) rats and (ob/ob) mice (Turnbaugh et al., 2006; Waldram et al., 2009; Palmnäs et al., 2020). They are also characterized by difference in abundance of *Firmicutes, Bacteroidetes*, and *Actinobacteria* (Turnbaugh and Gordon, 2009). Calorie restriction in obese individuals resulted in decrease in *Firmicutes/Bacteroidetes* ratio which were higher at baseline (Ley et al., 2006; Karlsson et al., 2013). In children, low *Bifidobacterium* abundance is generally linked to obesity (Abenavoli et al., 2019). Additionally, a gut microbiome dominated primarily by *Firmicutes* revealed altered methylation in gene promoters associated with obesity and cardiovascular disease (Kumar et al., 2014).

### Smoking

In comparison to controls, human microbiome studies on tobacco smokers revealed a lower relative abundance of *Bacteroides*, a higher relative abundance of *Prevotella*, and a lower Shannon diversity (Stewart et al., 2018). Following smoking cessation, decrease in *Bacteroides* along with changes in abundance of alphaproteobacteria and betaproteobacteria were observed (Nolan-Kenney et al., 2020). In fact, quitting smoking caused significant changes in the gut microbiome, with an increase in *Firmicutes* and *Actinobacteria* and a decrease in *Bacteroidetes* and *Proteobacteria*. It also results in increase in microbial diversity (Biedermann et al., 2013). Importantly, when provided similar meals to prevent nutritional impacts, different gut microbiota composition was found in smokers and non-smokers (Kobayashi and Fujiwara, 2013).

### Exercise

There is mounting evidence that exercise can influence the human gut microbiome by altering the composition and function of gut microbiota (Clarke et al., 2014; Bressa et al., 2017; Barton et al., 2018; Durk et al., 2019). Matsumoto et al. (2008) discovered that butyrate synthesis of bacteria, like *Bifidobacteria*, increased after 5 weeks of exercise training in rats. In contrast to sedentary controls, women who exercised at least three hours per week had higher concentrations of *Faecalibacterium prausnitzii, Roseburia hominis*, and *A. muciniphila* (Bressa et al., 2017). Professional rugby players gut microbiota exhibited a higher relative abundance of 40 different bacterial taxa, increase in alpha diversity as well as reduced abundance of *Lactobacillus* and *Bacteroides* sp. as compared to lean sedentary group (Clarke et al., 2014). There is currently conflicting data regarding the relationship between exercise and the gut microbiome. For instance, some research on rodents found that exercise decreased the *Firmicutes* to *Bacteroidetes* ratio (Mika et al., 2015; Denou et al., 2016), whereas others found that exercise raised the ratio (Kang et al., 2014; Lambert et al., 2015).

## Mechanisms of bidirectional communication between gut and brain

Gut-microbiota modulates brain functions by stimulating neuronal responses such as VN or secreting metabolites that directly control brain behavior. The gut microbiota communicates with the brain through the nervous (VN), endocrine (HPA axis), immune, and metabolic systems. The gut communicates with the brain via two pathways: Direct gut-brain communication is mediated through ANS and the spinal cord. Communication between gut and brain happens through ENS in the gut and ANS; and VN in the spinal cord.

### Gut-microbiota to brain interaction

Although the gut microbiota can interact through immunological and endocrine pathways, the VN signaling is the quickest and most direct route for the microbiota to influence the brain. The 10th cranial nerve, or VN, connects the viscera to the brain. It is a paired nerve made up of sensory and motor neurons (afferent and efferent, respectively). Vagal efferent transmit messages “down” from the brain to the gut through efferent fibers, which make up 10–20% of all fibers, whereas vagal afferents transmit signals “up” from the intestinal wall to the brain, making up 80–90% of all fibers (Tubbs et al., 2015). The activation and regulation of the HPA axis, which regulates the adaptive responses to stressors, is mediated by the vagal afferent pathways (Tsigos and Chrousos, 2002). EECs, 1% of intestinal epithelial cells, communicate with vagal afferents either directly by releasing serotonin that activates 5-HT3 receptors on these fibers or indirectly through the action of gut hormones that target the brain, such as cholecystokinin (CCK), glucagon-like peptide-1, and peptide YY, via vagal afferents that express receptors for these anorexigenic or orexigenic (ghrelin, orexin) hormone (Strader and Woods, 2005). In addition to cell-mediated sensing, VN has direct mechanisms for sensing microbial signals. For example, depending on the compound, SCFAs activate vagal afferent fibers through multiple ways. For example, butyrate directly affects afferent terminals, but the long fatty acid oleate acts on vagal afferents via a CCK-mediated mechanism (Lal et al., 2001). Additionally, TLR4 is also expressed on the vagal afferent fibers, which can detect bacterial products like Lipopolysaccharide (LPS) and stimulate the brain (Goehler et al., 1999). LPS acts on TLR4 and initiates an inflammatory response to release various pro-inflammatory cytokines and chemokines (Peña et al., 2014; Covington et al., 2015). The team of Mark Lyte used c-fos as a marker of neuronal activation to map brain circuits activated by oral administration of *Campylobacter jejuni* at subclinical concentrations in mice, impacting behavior and brain functions, highlighting the indirect stimulation of vagal afferent fibers by microbes (Gaykema et al., 2004). Both, the nucleus tractus solitarius (NTS), the first entrance point of vagal afferents in the brain, and the NTS’s widespread projections exhibited the signs of brain activation. Chronic administration of *L. rhamnosus* in rodent species showed increased GABA_B_ receptor expression in the prefrontal cortex, which is crucial for anti-depressant activity. *L. rhamnosus* is also responsible for reducing stress-induced corticosterone levels indicating the influence of *Lactobacillus* on CNS has significant impact on physiological levels. The absence of neurochemical and behavioral benefits of *L. rhamnosus* in vagotomized mice identifies the vagus as a significant modulatory constitutive communication channel between the gut and the brain (Bravo et al., 2011).

### Brain to gut-microbiota interaction

The composition and overall biomass of the intestinal microbiota are modulated by different types of psychological stressors. Short term stressors can also have an influence on the microbiota, with social stress exposure lasting only 2 h having a considerable impact on the community profile and a reduction in the relative proportions of the major microbiota phyla (Galley et al., 2014). Neurons, immunological cells, and ECC secrete signaling molecules under the control of the brain, which may have direct impact on the microbiota. According to a number of studies, bacteria have binding sites for enteric neurotransmitters produced by the host and can affect how certain components of the microbiota operate, which can increase susceptibility to inflammatory and infection stimuli (Hughes and Sperandio, 2008). *P. fluorescens* has been found to have high affinity for the GABA system and binding characteristics that are similar to those of brain receptors (Guthrie and Nicholson-Guthrie, 1989). Escherichia coli O157:H7 has a receptor for host-derived epinephrine/norepinephrine that can be selectively inhibited by adrenergic antagonists (Clarke et al., 2006). Additionally, the brain plays a significant role in the regulation of gut functions like motility, secretion of acid, mucus, and bicarbonates, handling of intestinal fluids, and the mucosal immune response, all of which are crucial for maintaining the mucus layer and biofilm, where various bacterial species grow in a variety of different microhabitats and metabolic niches linked to the mucosa (Macfarlane and Dillon, 2007). The disruption of the normal mucosal environment caused by a dysregulation of GBA can therefore have an impact on the gut microbiota. The quantity and quality of mucus vary in response to stress (Rubio and Huang, 1992). Acoustic stress has an impact on dogs’ intestinal and gastric postprandial motility, prolonging the recovery of the migratory motor complex pattern and temporarily reducing gastric emptying (Gué et al., 1989). Through the central release of CRF, mental stress also increases the frequency of cecocolonic spike-burst activity (Gue et al., 1991).

By altering intestinal permeability, which allows bacterial antigens to pass through the epithelium and trigger an immune response in the mucosa, the brain might also have an impact on the composition and function of the microbiota. Acute stress increased colonic paracellular permeability by increasing interferon-γ and downregulating ZO-2 and occluding mRNA expression (Demaude et al., 2006). The brain might also regulate immune function via the ANS. The sympathetic branch controls the quantity, degranulation, and activity of mast cells, resulting in an imbalance in tryptase and histamine release in stress-related muscular failure (Santos et al., 1998). It is crucial to note that gastrointestinal changes brought on by stress promote the production of pathogenic bacteria. The expression of *P. aeruginosa* is induced by norepinephrine produced during surgery, which might cause gut sepsis (Alverdy et al., 2000). Additionally, norepinephrine may promote the overgrowth of both pathogenic (*E. coli* 0157:H7:3) and non-pathogenic *E. coli* isolates (Freestone et al., 2002, 2003), as well as increase the virulence of *Campylobacter jejuni* and other intestinal pathogens (Cogan et al., 2007).

### Hypothalamus pituitary adrenal axis

The HPA axis is essential for the species’ survival. It is one of the critical non-neuronal communication routes in the microbiota-gut-brain axis (mGBA). Upon disturbance in homeostasis, there is a release of corticotrophin-releasing factor (CRF) and arginine vasopressin (AVP), a nonapeptide, from the hypothalamus into the hypophysial bloodstream, which stimulates adenohypophysis of pituitary gland thus trigger the release of ACTH into systemic blood circulation. In adrenal glands (also known as supra renal glands), the released ACTH binds to the receptors in zona fasciculata (adrenal cortex) and stimulates the steroidogenic pathway by facilitating the conversion of cholesterol esters into free cholesterol. Upon a series of enzymatic reactions, the cholesterol is converted into various steroidal end products, the glucocorticoids, i.e., cortisol in humans and corticosterone in rodents. These freshly synthesized glucocorticoids are rapidly released into blood circulation. The released glucocorticoids bind to the glucocorticoid receptor (GR) present in the cytoplasm, and the glucocorticoid-receptor complex translocate into the nucleus and binds to DNA. Thus, it acts as a transcription factor and results in target genes’ transcription, leading to protein synthesis changes (Fulford and Harbuz, 2005). The negative feedback regulation of the HPA axis is governed by two mechanisms, rapid and delayed feedback. Glucocorticoids exert the rapid feedback at the level of the hypothalamus by inhibiting the synthesis and release of ACTH. Delayed feedback at the level of adenohypophysis where glucocorticoids inhibit the mRNA expression of pro-opiomelanocortin (POMC), the ACTH precursor protein (Fulford and Harbuz, 2005).

Hypothalamus pituitary adrenal axis interacts with various neuronal and non-neuronal pathways of communication between the gut and the brain. It is found that VN stimulation results in a rise in plasma ACTH and corticosterone levels in rodents by increasing the CRF mRNA expression in the hypothalamus. Increased levels of cortisol induce an inflammatory cytokine-mediated surge in anxiety, bowel movement, and intestinal permeability while altering intestine’s microbiota (Wallace and Milev, 2017).

## Gut-brain axis regulating hypothalamus pituitary adrenal activity

The relationship between the HPA axis and the gut microbiota has been explained by a number of different mechanisms. First, Increased cytokine release and the production of small bioactive molecules because of the gut microbiota dysbiosis can cause certain cytokines, such as interleukin (IL)-1, IL-6, and tumor necrosis factor (TNFα) to pass through the BBB and act as potent HPA axis activators (Turnbull and Rivier, 1995; Banks, 2005). Second, the release of LPS and peptidoglycan, components of bacterial cell wall, can also activate the HPA axis (Arentsen et al., 2016). Third, the ClpB protein (mimics α-melanotrophin MSH) produced by *E. coli* stimulate the release of POMC thus promote ACTH synthesis (Breton et al., 2016). GF animal studies suggested that gut microbiota plays a significant role in the development and modulation of the HPA axis. Experimental studies in GF mice showed increased HPA axis activity and higher level of corticosterone when exposed to mild restraint stress as compared to SPF mice (Sudo et al., 2004). Brain derived neurotropic factor (BDNF) expression level was also decreased in the cortex and hippocampal region of GF mice compared with SPF mice (Sudo et al., 2004). This experiment demonstrates that vegetation of gut microbiota composition in early life is critical for the appropriate development of the HPA axis and stress response. Bacterial metabolites such as LPS can act on TLR4, which then initiates cytokine release and is responsible for stimulating the release of hypothalamic CRH. LPS can also directly stimulates adrenal glands to secret cortisol in humans and corticosterone in animals (Zacharowski et al., 2006). Several cytokines such as TNF-α and IL-12, are responsible for elevated corticosterone levels in animals.

Patients with different phases of mood and psychotic disorders can show disruption of the HPA axis. The precise mechanisms driving these findings are still unknown. Various studies have demonstrated a link between the composition of the gut microbiota and the HPA axis activity. Different stresses may affect intestinal integrity and abundance of *Bacteroides, Lactobacilli*, and *Clostridium* in animal models (Qu et al., 2021). Probiotics based on *Lactobacillus* and *Bifidobacterium* have also been shown to ameliorate depression- and anxiety-like symptoms, enhance learning, and restore stress-induced HPA axis dysfunction (Desbonnet et al., 2010). Furthermore, there is evidence that *Lactobacillus farciminis* supplementation in rats may dampen the HPA axis response to partial restraint stress by decreasing gut permeability (Ait-Belgnaoui et al., 2012). SCFAs have also been demonstrated to reduce the expression of genes that code for proteins involved in HPA axis (van de Wouw et al., 2018). Besides, maternal separation in animals creates an early life stress event, leading to an altered gut-brain axis and increases plasma corticosterone levels in the maternally separated animals. The maternally separated animals showed a higher immunological response and increased HPA activity (O’Mahony et al., 2009).

## Gut-brain hypothalamus pituitary adrenal axis dysregulation in depression

Depression is a mood-related disorder characterized by the depletion of monoamines such as serotonin, nor-adrenaline, and dopamine in the midbrain and nuclei of the brainstem. Several factors responsible for the cause of such disease include genetic factors, environmental factors, hyperactivity of the HPA axis, poor diet, low-grade inflammation, gut microbiota alteration, vitamin D deficiency, psychosocial stressors, etc. (Berk et al., 2013; Jokela et al., 2016). The proposed hypothesis suggests that increased CRH is secreted by neuronal cells of the paraventricular nucleus of the hypothalamus in patients suffering from depression (Raadsheer et al., 1994). CRH stimulates the pituitary gland to secrete ACTH, which further activates the release of corticosteroids from adrenal glands (Barden, 2004). An impaired feedback mechanism is also found in depressive patients who cannot control the excessive release of CRH and ACTH. Hypercortisolemia is generally seen in these patients, and further leads to a decrease in the sensitivity of GR (Liang et al., 2018). To overcome the stress response, the levels of ACTH and corticosterone are elevated in microbiota deficient mice compared to SPF mice (Sudo et al., 2004). Anhedonia and anxiety-like response are observed after pooled FMT from depressed patients than FMT from healthy rats (Kelly et al., 2016). All these results suggest the direct relationship between gut microbiota and the HPA axis.

Various studies conducted so far suggest the involvement of gut microbiota in the modulation of depression-like behavior, including pre/probiotic treatment, GF mice, antibiotic-induced gut microbiota alteration, FMT, and deliberate microbial contamination of GIT (Bravo et al., 2011; Messaoudi et al., 2011; Cryan and Dinan, 2012; Desbonnet et al., 2015; Wong et al., 2016; Gur et al., 2017). In recent studies, certain species of gut-microbiota such as *Prevotella* (type 2), *Bacteroides* (type 1), and *Proteobacteria* were also found to be increased and caused dysbiotic conditions in patients suffering from depression (Liu et al., 2016; Evrensel et al., 2020). These micro-organisms are responsible for the dysbiotic states, cause a leaky gut, and translocation of bacterial metabolites into systemic circulation. This further activates pro-inflammatory cytokinin such as IL-1, IL-1β, IL-6, and TNF-α, which promotes the activation of microglia and astrocytes (Song and Wang, 2011). Cytokines such as IL-1 are responsible for activating neurons in the paraventricular nucleus, releasing CRH, and promoting a chronic increase in blood corticosteroid level (Song et al., 2009). Patients suffering from depressive disorder were found to have decreased number of protective microbial species like *Bifidobacterium* and *Lactobacillus* in their intestinal flora, which may be a key factor for dysbiotic conditions and initiation of the inflammatory process. Probiotic treatment of these strains was effective in disease conditions (Aizawa et al., 2016; Evrensel et al., 2020). The probiotic therapy of *L. Rhamnosus* showed improvement in mood and behavior by decreasing the elevated level of corticosterone in a stress-induced depressive model of rodents (Bravo et al., 2011). The pharmacological anti-depressant-like effect of (R)-Ketamine, a dissociative hallucinogen, NMDA receptor antagonist, and as an antidepressant; is altered because of the dysbiotic gut microbiome in the chronic social defeat stress model of mice (Qu et al., 2017; Yang et al., 2017). Huang et al. (2019) revealed the role of phylum *Actinobacteria* and the class *Coriobacteriia* in the anti-depressant effect of ketamine. Furthermore, FMT from alcoholic individuals induced depression-like behavior, spontaneous alcohol preference as well as decreased BDNF, α-1 subunit of GABA A receptor (α-GABA_A_ R) in medial PFC; and glutamate receptor 1 (mGluR1) and protein kinase C ε in NAc in C57BL/6J mice (Zhao et al., 2020). Contrarily, FMT from healthy individuals decreased alcohol-induced depressive behavior in mice (Xu et al., 2018). Another study on FMT from anhedonia-susceptible rats revealed exaggerated pain and depression-like behavior in antibiotic-treated pseudo-GF mice. In contrast, transplantation from resilient rats exhibits significant improvement in pain and depressive-like behavior (Yang et al., 2019). This signifies that gut microbiome, dysbiotic condition, and inflammation can be the underline cause of depression. Specific strains of prebiotic or probiotic therapy of gut microbiota can be the key factors in the treatment of depression.

## Gut microbiome mediated epigenetic modifications

**FIGURE 2:**
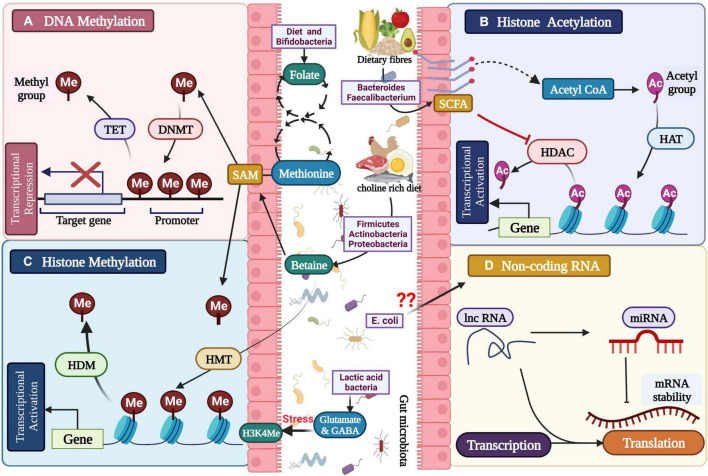
Gut microbial metabolites regulating the epigenetic mechanisms: **(A)** DNA methylation, **(B)** histone modification, **(C)** histone methylation, and **(D)** non-coding RNA associated gene silencing.

For millions of years, the gut microbiota has coevolved and works in symbiotic association with humans. These prokaryotic microscopic members of the ‘human holobiont’ play a key role in maintaining normal physiology and homeostasis. Aberration in gut microbiome configuration and function is linked to being a causative factor for the development of various neurological diseases such as Alzheimer’s disease, Parkinson’s disease, depression, etc. These micro-organisms follow multiple complex molecular mechanisms for maintenance of normal homeostasis such as vagal nerve signaling (Berk et al., 2013) neuroendocrine signaling (Jokela et al., 2016) metabolic signaling (Bravo et al., 2011), immune system modification (Cryan and Dinan, 2012), and epigenetics (Abe-Higuchi et al., 2016; Higuchi et al., 2016). Epigenetics has a crucial role in regulating host physiology via alteration in gut microbiome metabolic activity, which depends on environment and diet. The metabolites produced by gut microbiota act as cofactor and substrate for various enzyme reactions; for example, cofactors for the activity of enzyme acetylases and methylases, which regulate histone modification and DNA methylation; comes from the gut microbiome (Figure 2)(Cai et al., 2015; Ledford, 2015; Abe-Higuchi et al., 2016). Regulation of epigenetics is a dynamic process and is subject to changes in exercise, nutrition, and microbiota composition (Libert et al., 2011).

The literal meaning of epigenetics is “in addition to genetics.” It involves studying gene expression changes in the chromosome instead the DNA sequence. These changes are heritable as well as stable. Changes in Chromosomal superstructure and chemical modification of nitrogenous bases are mainly regulated by epigenetics without directly altering the DNA sequence. Various molecular mechanisms can lead to epigenetics, but the main pathways include DNA methylation and acetylation, histone modification, and RNA-associated silencing.

### DNA methylation

Methylation of DNA occurs covalently to the pyrimidine ring of cytosine, which results in a change in structure of major grooves. The methyl group is extracted from S-adenyl methionine (SAM). The enzymes DNA Methyltransferases (DNMTs) catalyze DNA methylation by transferring the methyl group to carbon-5 of cytosine and forming 5-methyl cytosine. There are three major DNMTs in mammals viz, DNMT1, DNMT3a, and DNMT 3b. DNMT3L is catalytically inactive, but can increase the activity of Dnmt3a/b to 15-fold upon binding with them. DNMT3a and DNMT3b are *de novo* DNMT because they establish new methylation patterns for unmodified DNA (Gowher et al., 2005). Cytosine is the most common site for methylation, followed by guanine nucleotide and CpG (5′-C-phosphate-G-3′) rich sites. The CpG-rich region in the DNA is called CpG Island. These DNMTs are extremely sensitive to the availability of nutrients, which can be influenced by the metabolic activities of the gut microbial species. Various metabolites such as cyanocobalamin, choline, folate, and betaine play an essential role in synthesizing 6-methyltetrahydrofolate, which acts as a methyl group donor for SAM that is directly involved in the DNA methylation process (Covington et al., 2009; Hunter et al., 2009). These metabolites are regulated by specific folate-producing gut microbiota such as *Bifidobacteria* and *Lactobacillus* (Otsuki et al., 2008; Uchida et al., 2011). DNA methylation plays a vital role in the inactivation of the X chromosome (Bosker et al., 2011; Hooper et al., 2012; Sun et al., 2013), regulation of tissue-specific gene expression, silencing of retroviral elements and genomic imprinting (Sullivan et al., 2000).

Pieces of evidence are available about methylation of various genes in depression. Increased CpG methylation in the BDNF gene’s promoter region has been linked to a reduction in the amount of BDNF produced by neurons (Martinowich et al., 2003). In mouse model of depression, epigenetic modifications result in alteration of chromatin structure of BDNF (Tsankova et al., 2006). Additionally, majority of depressed population exhibits increased DNA methylation on BDNF gene (Fuchikami et al., 2011). These results suggested that BDNF epigenetic alterations may be crucial to pathophysiology of depression and to its potential therapeutic use. Majority of the studies conducted so far, to establish the correlation between depression and DNA methylation of BDNF, revealed hypermethylation of BDNF gene in depressed patients compared to healthy individuals (Fuchikami et al., 2011; Kang et al., 2015). Contrarily, one study in pregnant women have associated depressive symptoms with reduced methylation of BDNF promotor in offspring (Braithwaite et al., 2015).

In humans SLC6A4, serotonin transporter, paly key role in development and function of brain region (Booij et al., 2013). DNA methylation has received a lot of attention recently in studies, and this focus may help to explain how the regulation of SLC6A4 gene expression affects changes in emotional behavior. Lower SLC6A4 mRNA levels were linked to higher methylation in human lymphoblast cell lines (Philibert et al., 2007), suggesting that SLC6A4 promoter methylation may be linked to gene expression. Additionally, it was discovered that SLC6A4 promoter methylation, both full and partial, dramatically decreased SLC6A4 expression levels (Olsson et al., 2010). Numerous studies have revealed association between SLC6A4 DNA methylation and depression (Philibert et al., 2007; Devlin et al., 2010; Olsson et al., 2010; Zhao et al., 2013). The majority of them demonstrated an increase in SLC6A4 DNA methylation in depressive disorders. Three studies, however, discovered that there was no variation in the DNA methylation of SLC6A4 between depressive patients and healthy controls (Olsson et al., 2010; Bayles et al., 2013; Chagnon et al., 2015). On the other hand, depression disorders are also linked to DNA methylation in the serotonin receptor (5-HTR) family (Perez-Cornago et al., 2014; Weder et al., 2014; Perroud et al., 2016). According to research by Perez-Cornago et al. (2014), a decrease in depression symptoms was associated with an increase in the DNA methylation of the 5-HTR2A gene. In contrast, Perroud et al. (2016) discovered that in bipolar disorder patients who attempted suicide, the level of 5-HTR3A methylation was higher at CpG2 III and CpG4 III and lower at CpG1 I and CpG5 III. Additionally, other genes related to the metabolism of 5-HT may be crucial in the development of depression. Three experiments with MAO-A, which catalyzes the oxidative deamination of 5-HT, revealed variable DNA methylation levels. Among 43 CpG sites, Katharina Domschke discovered only two hypomethylation sites (Domschke et al., 2014). Melas PA observed that the DNA methylation level of the MAO-A gene exon 1 rose in depressed individuals (Melas et al., 2013). In contrast, Melas PA and Forsell found it decreased in female patients (Melas and Forsell, 2015). In conclusion, numerous investigations have found a link between SLC6A4 methylation and depression. To further understand the involvement of epigenetic mechanisms of SLC6A4 methylation in the etiology of depression, more research in the field of epigenetics is required.

The GR, which is encoded by the protein-coding gene NR3C1 (Nuclear Receptor Subfamily 3, Group C, Member 1), is activated by cortisol (Francke and Foellmer, 1989). GR (ligand activated transcription factor) is necessary for the HPA axis to operate properly. Individuals with GR anomalies in their brains were found to be connected to bipolar disorder and schizophrenia. It has been suggested that the genetic variants in the GR (NR3C1) gene may be the mechanism causing HPA axis malfunction and GR abnormalities in psychiatric disorders (Turner et al., 2010). In particular, it has been shown that NR3C1 methylation contributes significantly to our understanding of both depression and dysregulation of the HPA axis. In three investigations, it was discovered that new-born of depressive mothers had higher NR3C1 DNA methylation levels (Oberlander et al., 2008; Conradt et al., 2013; Murgatroyd et al., 2015). However, Braithwaite et al. (2015) found that this rise exclusively affected male new-born. In addition, female depressive individuals with early parental deaths had hypermethylation. These findings imply that depression can have a gender-specific impact on the DNA methylation of the NR3C1 gene. Comparing NR3C1 methylation to controls in more recent research, however, did not reveal any correlation between the two conditions (Alt et al., 2010; Kim et al., 2016). Contradictory results were found in other research comparing depression to controls, with either increased (Melas et al., 2013) or decreased (Na et al., 2014) level of DNA methylation. According to some studies, the DNA methylation of FK506 binding protein (FKBP5), a co-chaperone of the GR, increased in depressed patients (Weder et al., 2014; Höhne et al., 2015) indicating a connection between depression and the DNA methylation in the GR system. Undoubtedly, there is conflicting and muddled evidence supporting the relationship between NR3C1 methylation and depression, the reason being small sample size, various tissue types, low effect size, different ethnicities etc. Therefore, prior to conducting the investigations, future research should take these factors into account.

Several physiological and behavioral functions, including the operation of the HPA axis, are regulated by the neuropeptide oxytocin, which is released by the paraventricular nucleus of the hypothalamus. Recent research suggests that the polymorphisms of the oxytocin receptor (OXTR) affect the neurocardiac response to the HPA function (Norman et al., 2012), suggesting that OXTR may have regulatory effects on the pathomechanisms of depression. There is evidence of OXTR CpG hypermethylation in depressed women (Bell et al., 2015; Chagnon et al., 2015; Reiner et al., 2015). However, DNA hypomethylation was observed in depressed African American cohort (Smearman et al., 2016). Contrarily, Kimmel et al. (2016) did not find any discernible genetic effects.

### Histone modification

Histones are the core proteins which give the backbone to chromatin. DNA wraps around histones to form a nucleosome. The nucleosome consists of pair of histones (H2A, H2B, H3, and H4) at the core, around which DNA is tightly bound. The histone modification types include acetylation, methylation, phosphorylation, biotinylation, citrullination, SUMOylation, ubiquitination, and proline isomerization. Among all the mentioned modifications, methylation, acetylation, and deacetylation are the most common (Kendler, 1983; Sun et al., 2013). The N-terminal of H3 usually undergoes histone modification via methylation or acetylation of lysine and arginine residue, whereas phosphorylation occurs at serine and threonine residues (Tsankova et al., 2007). Methylation involves the addition of methyl groups to the histone proteins by histone methyl transferases (HMTs). The methylation of histone can lead to transcription activation [methylation of H3 lysine 4 (H3K4)] or transcription inactivation [methylation of H3 lysine 9 (H3K9) and H3K27] depending upon the specific residue (Peña et al., 2014; Yamagata et al., 2017). Acetylation of histones results in neutralization of N-terminal, leading to a reduction in affinity for DNA and loosening of chromatin conformation, thus leads to transcription activation (Fraga et al., 2005). In histone acetylation, the acetyl group from acetyl CoA is transferred to terminal lysine residue by Histone acetyltransferase (HATs). Various metabolites from the gut microbiome, such as SCFA, are proved to regulate the histone acetylation process (Hobara et al., 2010; Sarkar et al., 2014). Histone deacetylases (HDACs) remove the acetyl group from the terminal lysine residue, which favors chromatin compaction and results in transcription inactivation (Iga et al., 2007). Total of 13 HDACs are found in humans and are mainly classified into four classes: Class I includes HDACs 1, 2, 3, and 8 (most similar to yeast transcription regulator RPD3); Class II contains HDACs 4, 5, 7, and 9; Class IIb consist of HDACs 6 and 10. The class II HDACs are similar to yeast deacetylase, HDA1.; Class III includes Sirt1 to Sirt7, which deacetylates histone as well as non-histone proteins, and Class IV comprised of a single member, HDAC 11, which does not share any similarity with RPD3 and HDA1 (Xu et al., 2018; Zhao et al., 2020). HDACs are found to be overexpressed in various neurological and inflammatory disorders. The SCFAs produced by the gut microbiome are involved in HDAC inhibition; butyrate is a specific HDAC Class I and II inhibitor (Higuchi et al., 2016).

Gram-positive, anaerobic bacteria belonging to the genera *Coprococcus* and *Faecalibacterium* digest dietary fibers to produce SCFAs. One of the most prevalent genera of gut microbes, *Faecalibacteria* have significant immunological roles as well as therapeutic importance for a number of disorders, including depression (Jiang et al., 2015). SCFAs can bind to and activate G protein-coupled receptors GPR43/41 (FFAR2/3), as well as the less prevalent GPR109a and CPR164 (also known as OR51E1 and HCAR2, respectively). Since these receptors are widely expressed in many different human organs, such as EEC, adipocytes, immune cells, and neurons, it is possible that SCFAs can change behavior either directly by stimulating neuronal pathways or indirectly by activating neuroendocrine and immunological systems (Stilling et al., 2016). With regard to depression, lactate, one of the SCFA, has both preventive and reversible effects, and these effects are carried out on HDACs by distinct epigenetic mechanisms (Karnib et al., 2019). The social avoidance and anxiety behaviors that resulted from the 10-day social defeat task were prevented in control mice by chronic lactate administration before the challenge. The class I HDAC2/3 level and activity were increased in lactate treated mice. After the onset of depression, the effect of lactate was not mediated by HDAC2/3 but rather by a decrease in HDAC5 levels (Karnib et al., 2019). A lack of butyrate, acetate, and propionate was observed in MDD patients (Chen et al., 2015; Jiang et al., 2015; Zheng et al., 2016; Skonieczna-żydecka et al., 2018), and a high number of butyrate-producing bacteria, such as *Faecalibacterium* and *Coprococcus* sp., was found in subjects with greater quality of life indicators (Valles-Colomer et al., 2019), supporting SCFA involvement with the etiology of depression.

### Non-coding RNA associated silencing

Non-coding RNA-associated silencing is a new epigenetic mechanism. Non-coding RNA (ncRNA) are nothing but RNA that is not transcribed into protein. They are classified into two types: housekeeping ncRNA and regulatory ncRNA. Based on the size, regulatory ncRNA are further divided into short chain ncRNA and long ncRNA (lncRNA). The short chain ncRNA includes small interfering RNA (siRNA), microRNA (miRNA) and piwi RNA (piRNA) (Desbonnet et al., 2015; Gur et al., 2017). Several studies have indicated the role of siRNA in gene silencing through DNA methylation and histone modification (Messaoudi et al., 2011; Wong et al., 2016; Qu et al., 2017). The direct role of miRNA in epigenetics has not been reported in mammalian cells. However, few studies suggest the change in whole DNA or chromatin state by miRNA by inhibiting the activity of chromatin remodeling enzymes (Yang et al., 2017; Huang et al., 2019). Several miRNAs were found to be dysregulated in depression. MiR-124, miR-139a-5p, miR-221, miR-218, miR-17-5p, miR-146a, miR-132, miR-425-3p, miR-184 were some of the miRNAs identified in various biological samples of depression patients as well as in preclinical models (Shi et al., 2021). Several miRNAs derived from the host exosomes play an important role in maintaining the gut microbiota and the host physiology. The importance of miRNAs in the maintenance of gastro-intestinal functions is also widely recognized (Zhao et al., 2021). But the relation between the gut dysbiosis induced miRNA dysregulation in depression has not been established. Future studies aimed at exploring this association might provide novel insights into pathogenesis as well as treatment of depression.

## Evidence of epigenetic changes in depression

The epigenetic modifications play a key role in anti-depressant response as well as in the pathophysiology of depression (Tsankova et al., 2007; Sun et al., 2013; Peña et al., 2014; Yamagata et al., 2017). The levels of acetylated histones in the limbic region were affected by chronic stress (Covington et al., 2009; Hunter et al., 2009). Six weeks of chronic stress results in depressive behavior in mice by increasing the HDAC2 function in the ventral striatum (Uchida et al., 2011), indicating HDAC2 involvement in stress vulnerability, thus favoring the anti-depressant potential of HDAC2 inhibitors. Glial-derived neurotrophic factor (GDNF) level gets lowered in the ventral striatum in response to chronic stress, which is prevented by knockdown of HDAC2, suggesting that GDNF is a primary target of HDAC2. There is evidence of high HDAC2 expression and low GDNF expression in depressed patients (Otsuki et al., 2008; Hobara et al., 2010).

Similarly, HDAC4 and HDAC5 are also overexpressed in depression (Iga et al., 2007; Otsuki et al., 2008; Sarkar et al., 2014). A previous study has reported that hippocampal administration of HDAC4/5 inhibitors prevented chronic stress-induced depressive behavior (Higuchi et al., 2016), which can be considered a new treatment strategy for depression. Following chronic stress, SIRT1, a class III HDAC shows reduced activity in the dentate gyrus. Increased depressive behavior is observed after the blockade of hippocampal SIRT1 activity either with drugs or through a genetic approach. Contrarily, during chronic stress, hippocampal SIRT1 activation results in the blockade of depressive behavior and abnormal dendritic structures and increased phosphorylation of extracellular signal-regulated protein kinases 1 and 2 (ERK1/2). ERK2 overexpression in the hippocampus has an anti-depressant effect, whereas lower expression is associated with increased depressive symptoms, indicating the critical role of SIRT1 in regulating depression (Abe-Higuchi et al., 2016). SIRT1 exhibit a significant genome-wide association with MDD (Cai et al., 2015; Ledford, 2015). Single nucleotide polymorphism (SNP; rs12413112) in SIRT1 is associated with MDD (Libert et al., 2011). There are number of drugs with the epigenetic mechanism of action beneficial to counteract depressive symptoms and are listed in Table 2.

**TABLE 2:** Epigenetic drugs in depression.

Sr. no	Drug	Route of administration	Animal	Role/Mechanism	Target	Findings	References
1.	MS275	Through osmotic minipump for 14 days	C57BL/6J mice	HDAC inhibitor	Class I HDAC	HDAC2 inhibition exerts anti-depressant like effect by increasing H3 acetylation in nucleus accumbens and PFC	Covington et al., 2015
2.	Cpd60	Intraperitoneal (i.p.) for 07 days	C57BL/6 mice	HDAC inhibitor	Selective HDAC 1 and 2	Exerts anti-depressant like effect by selective inhibition of HDAC 1/2	Schroeder et al., 2013
3.	LMK-235	Through osmotic minipump for 2/4 weeks	BALB/c mice	HDAC inhibitor	Selective HDAC 4/5	Exerts anti-depressant like effect	Higuchi et al., 2016
4.	SAHA (vorinostat)	Intravenous for 6 days	C57BL/6J and BALB/c mice	HDAC inhibitor	Class I and II HDAC	More rapid anti-depressant effect than imipramine or fluoxetine	Uchida et al., 2011
		i.p. single injection	Crtc1–/–		Hippocampal HDAC9 up-regulation	Partial anti-depressant like effect	Meylan et al., 2016
		Through osmotic minipump for 14 days	C57BL/6J mice			Exerts anti-depressant like effect	Covington et al., 2015
5.	Sodium butyrate	i.p. for 28 days	C57BL/6J mice	HDAC inhibitor	Class I and II HDAC	SB exerts anti-depressant like effect	Schroeder et al., 2007
		i.p. for 14 days	ICR mice		HDAC 2/5	SB exerts anti-depressant like effect	Han et al., 2014
6.	SRT2104	Bilateral hippocampal osmotic minipump for 16 days	BALB/c mice	SIRT1 activator	SIRT 1	Exerts anti-depressant like effect	Abe-Higuchi et al., 2016
7.	33i	i.p. for 3 weeks	VGLUT1 (VGLUT1+/–) mice	SIRT2 inhibitor	SIRT2	Exerts anti-anhedonic effect	Muñoz-Cobo et al., 2017
		i.p. for 10 days/2 weeks	C57BL/6J mice			33i increases serotonin levels and glutamate receptor subunits.	Erburu et al., 2017
8.	Zebularine	In NAc through osmotic minipump for 4 weeks	BALB/c mice	DNMT inhibitor	DNMT‘	Exerts anti-depressant like effect	Uchida et al., 2011
9.	RG108	In NAc through osmotic minipump for 4 weeks	BALB/c mice	DNMT inhibitor	DNMT	Exerts anti-depressant like effect	Uchida et al., 2011
		i.p. single injection	Swiss albino mice			Exerts anti-depressant like effect	Sales and Joca, 2015
		Continuous intra-NAc infusion over 7 days	Naïve mice			Exerts anti-depressant like effect	Laplant et al., 2010

## Interplay between gut-brain axis and epigenetics in depression

As discussed earlier, epigenetic modifications bring about changes in gene expression because of changes in the external or internal environment without altering the DNA sequence. These changes are stable for the long term, even if the exposure to that causative factor is for a short period. Microbiota is also one of the environmental factors that can alter host epigenome via GBA modification and results in visible behavioral or phenotypic changes. Though these changes are long-lasting but are not permanent and hence can be reversed at a later stage by various methods such as restoration of gut microbiome through pre and probiotic supplementation, FMT, and lifestyle modification approaches like proper sleep cycle, healthy eating habits, physical exercise, yoga, and meditation. All the above approaches are beneficial in diabetes, obesity, neurodegenerative diseases, and depression.

The microbial count of humans is 10 times higher than own human cells. The human gut comprises three to four million unique genes, which is 100–150 times more than our genome (Qin et al., 2010). These genes are responsible for the production of various proteins and metabolites of the microbiome. These metabolites directly or indirectly affect the host genome by modulating the host epigenome (Table 3).

**TABLE 3:** Interplay between the GBA and epigenetics in depression.

Microbial metabolite	Micro-organism implicated	Link to microbiota gut-brain axis	Link to epigenetics	Status in depression	References
Short-chain fatty acids (SCFA)	**Butyrate:** *Eubacterium rectale*, *Roseburia faecis*, *Eubacterium hallii*, and *Faecalibacterium prausnitzii* appears **Propionate:** *Veillonella* spp., *Lactobacillus* spp., *Bacteroides* spp., and *Propionibacterium* sp. **Acetate:** Numerous bacterial class	SCFA can cross blood brain barrier and modulate the levels of neurotropic factors, neurotransmitters, neurogenesis and reducing neuroinflammation and glia dysfunction thus play a key role in gut-brain communication. They can induce the production of GABA and serotonin and thus stimulate indirect signaling to the brain by interaction with EEC.	SCFAs, especially butyrate, acetate, and propionate have HDAC inhibitory activity and thus causes transcriptional activation by facilitating euchromatin confirmation.	SCFA’s are downregulated in depression. The abundance of *Faecalibacterium* and *Coprococcus* spp. is also lowered in the gut. SCFAs are also found to get lowered in depressed women compared to the non-depressed ones indicating its potential role in etiology of disease.	Kendler, 1983; Sullivan et al., 2000; Tsankova et al., 2007; Sun et al., 2013; Peña et al., 2014; Smith, 2014; Yamagata et al., 2017
Lactate	Lactic acid bacteria, *Bifidobacteria* or *Proteobacteria*	The energy requirement of the brain can be fulfilled by lactic acid as it can cross the BBB and influence various neuronal functions such as plasticity, excitability as well as memory consolidation.	Recent studies revealed that lactate has the ability to act as a precursor for lactylation of histone lysine residues, a novel epigenetic mechanism, and promote transcriptional activation from chromatin.	Lactate’s role in depression is not clear. Impaired mitochondrial function in a depression causes the accumulation of lactate in the brain as well as in the ventricles. There is evidence of the excretion of a high amount of lactate in urine in severely depressed patients as compared to their less depressed or non-depressed counterparts.	Hunter et al., 2009; Bosker et al., 2011; Uchida et al., 2011; Sun et al., 2013; Covington et al., 2015
Tryptophan (Trp) metabolites	**Synthesis:** trp is one of the essential amino acids, and few studies reported *E. coli* may synthesize trp using five genes present in trp operon **Metabolism:** **Phyla:** *Firmicutes, Bacteroidetes, Actinobacteria, Proteobacteria*, and *Fusobacteria* **Genera:** *Clostridium, Burkholderia, Streptomyces, Pseudomonas*, and *Bacillus*	Trp is a key amino acid in serotonin synthesis. Upon metabolism, it produces metabolites of the kynurenine pathway, tryptamine, indole acetic acid, indole butyric acid, etc, which act as signaling molecules for proper brain function	Various keywords were used to search the relationship between the tryptophan metabolite and epigenetics on different databases, but not a single study so far was available to see the epigenetic insight of the same. Thus, much research is needed to establish the connection between them as kynurenine pathway is a key pathway of depression.	Trp metabolism is up-regulated in depression which results in impaired serotonin synthesis, upregulation of indolamine 2,3 dioxygenase (IDO) activity, and 3-hydroxykynurenine and quinolinic acid which are neurotoxic in nature. Furthermore, there is a decrease in neuroprotective molecules such as kynurenine and kynurenic acid.	Iga et al., 2007; Otsuki et al., 2008; Hobara et al., 2010; Ledford, 2015; Abe-Higuchi et al., 2016; Higuchi et al., 2016
Amino acids	Numerous bacterial species participate in the synthesis and metabolism of several amino acids, including glutamate, phenylalanine, tyrosine, isoleucine, alanine, serine, and oxidized proline.	Several microbial populations’ especially lactic acid bacteria are involved in synthesizing glutamate and GABA, which are crucial neurotransmitters. Similarly, phenylalanine is also involved in the production of various catechol amines such as epinephrine, norepinephrine, and dopamine.	Lifestyle stressors are responsible for depression-like symptoms by stimulating the sympathetic nervous system, thus influencing the synthesis and release of neurotransmitters and stress hormones. Since these stressors are part of the environment, the involvement of epigenetic mechanisms is strongly believed to regulate gene expression. Regulation of GABAergic gene involves H3K4 methylation as well as histone modifications.	Phenylalanine, aspartate, glutamate, and serine are lowered in depressed individuals. Furthermore, impaired brain functioning of GABA, glutamate, and catecholamine is also reported. Various studies have revealed the critical role of gut microbiota in maintaining optimum serum and brain levels of numerous amino acids using GF and SPF mice.	Bravo et al., 2011; Cryan and Dinan, 2012; Berk et al., 2013; Desbonnet et al., 2015; Ledford, 2015; Jokela et al., 2016; Gur et al., 2017
Secondary bile acids (BAs)	Bacteria: *Clostridium, Enterococcus, Bifidobacterium, Lactobacillus, Bacteroides* and *E. coli*, *C. testosteroni*, and *Ruminococcus* sp. Archaea: *Methanobrevibacter smithii* and *Methanosphaera stadtmanae*	FXR and G-protein coupled bile acid receptor-1 (GPBAR-1) are responsible for bile acid signaling in the brain. Its brain signaling involves both direct (by crossing BBB) and indirect (through gut FXR and GPBAR-1 receptor activation) mechanisms for facilitating the synthesis of fibroblast growth factor 19 and glucagon-like peptide-1.	Various epigenetic transcriptional factors control the bile acid metabolism, such as *Brg-1* which interacts with FXR and promotes transcriptional activation of *SHP*. Other epigenetic transcriptional factors include *Brm, G9a, PRMT1, CARM1*, HDAC 1,2,3,7, P300 etc.	The FXR is the most prominent BA signaling pathway in neuropsychiatric disorders. In rats, up-regulation of FXR in the hippocampus is appeared to be involved in depression-like symptoms and reduced BDNF levels. Some anti-depressants, including paroxetine, has the ability to modify the gut microbiome and BA synthesis in mice.	Messaoudi et al., 2011; Wong et al., 2016; Qu et al., 2017; Yang et al., 2017; Xu et al., 2018; Huang et al., 2019; Zhao et al., 2020
Choline derived metabolites	*Firmicutes, Actinobacteria*, and *Proteobacteria*	Trimethylamine (TMA), betaine, phosphocholine, and acetylcholine (neurotransmitter) are the products of choline metabolism. The gut microbiome, TMA and TMA N- oxide (TMAO, metabolite of TMA) acts as an essential biomarker for choline metabolism as well as an indicator of a healthy brain.	Choline acts as a methyl donor for epigenetic regulation. In mice, the abundance of choline-consuming bacteria is responsible for aberrant global DNA methylation patterns.	High TMA and TMAO levels indicate gut dysbiosis and positively correlate with the severity of depression in males and females. Similarly, depressive and abnormal behavior is observed in mice having an abundance of choline consuming bacteria.	Sudo et al., 2004; Qin et al., 2010; Zhu et al., 2010; Kelly et al., 2016; Miro-Blanch and Yanes, 2019; Yang et al., 2019; Louwies et al., 2020
Estrobolome (The phrase describes a group of bacteria capable of metabolizing and regulating the body’s estrogen levels).	The circulating estrogen levels are greatly affected by the gut microbiome through various mechanisms, such as beta glucuronidase secretion. The level of non-ovarian estrogen is influenced by the gut microbiome, especially by *Clostridia* and members of *Ruminococcaceae* through enterohepatic circulation.	Gut microbiota might be involved in estrogen metabolism, thus influencing brain signaling. The neuroprotective effect of estrogen includes neuroplastic action in the hippocampus as well as regulation of serotonin receptor levels.	In case of cognition, the beneficial effect of estradiol is dependent on DNA methylation and histone acetylation. Contrarily, ER silencing is caused by DNA methylation in various types of cancer and HDAC1 and 3 might be involved in decrease expression of estrogen receptor alpha.	The estrogen metabolism is greatly affected by gut dysbiosis leading to low circulating estrogen levels. Many health conditions are associated with low circulating estrogen levels, including obesity, polycystic ovarian disorder, cognitive dysfunction, fertility, cardiovascular diseases, etc. The fact that three distinct types of depression are linked to the menstrual cycle, namely Premenstrual dysphoric disorder (PMDD), postpartum depression, and perimenopausal depression provides evidence that altered sexual hormones play a role in the pathophysiology of depression in women. In all the above-mentioned types of depression, estradiol seems to be effective compared to progesterone.	Ting and Trowsdale, 2002; Rossi et al., 2011; Kumar et al., 2018; Seeley et al., 2018; Sanders et al., 2019

Gut microbiota, for example, *Firmicutes*, produces SCFA by utilizing dietary fibers. These SCFA acts as HDAC inhibitors, thus promoting gene expression by blocking the removal of an acetyl group from histones (Zhu et al., 2010). The global histone acetylation (H3 and H4) and methylation (H3) along with chromatin modification were observed in a recent study in which C57BL/6 mice were supplemented with a mixture of SCFA (67.5 mM acetate, 40 mM propionate, and 25.9 mM butyrate) through drinking water (Louwies et al., 2020). Similarly, DNA and histone methylation are also regulated by gut microbiota by utilizing dietary methionine for the synthesis of SAM using the L-methionine *S*-adenosyl transferase (MAT) enzyme (Miro-Blanch and Yanes, 2019). SAM acts as a methyl donor for the transfer of methyl group to the 5th cytosine residue of the CpG Island by DNMTs and thus favors transcriptional repression. Ten eleven translocation (TET) enzyme functions for transcriptional activation through DNA methylation (Kumar et al., 2018). Folate, a methyl donor essential for the synthesis of SAM, is produced by *Bifidobacterium* sp. and *Lactobacillus plantarum*. Thus, gut dysbiosis can influence SAM levels, ultimately altering DNA and histone methylation state (Rossi et al., 2011).

Vagus nerve stimulation affects the hippocampal, cortical, and blood epigenetic transcriptomes in male Sprague Dawley rats and epigenetically modifies genes associated with neural plasticity and stress-response signaling (Sanders et al., 2019). Microbiota can cause an altered chromatin state, which might result in host immune maturation (Seeley et al., 2018). SCFAs are critical mediators of altered chromatin states. They regulate DNMTs and HDAC, thus regulating the expression of the MHC gene, which is involved in immune response (Ting and Trowsdale, 2002). According to a comparative NMR-based metabolome investigation using mouse models (Romano et al., 2017), there is a positive correlation between the luminal concentrations of gut metabolites and the number of Treg cells in the colon. Butyrate is a well-known anti-inflammatory molecule among SCFAs that promotes the differentiation of colonic Treg cells (Furusawa et al., 2013). Specific metabolite produced by gut microbiota acts as a cofactor for epigenetic reaction. In addition to being a necessary nutrient for the brain’s healthy development, choline also has a role in the production of SAM, a crucial methyl donor for the methylation of DNA and histones. Choline metabolizing bacteria competes with the host for dietary choline, thus resulting in low plasma and hepatic choline levels. Mothers with choline deficiency deliver fetus having hippocampal global DNA methylation and cholinergic neurons with altered function (Romano et al., 2017).

## Limitations and future perspective

The majority of studies so far has been on the bacteria that dwell in our gut, although various species have diverse homes and functions throughout the body. Finding the correct data from the microbiome, which is necessary to understand how it affects our health, is one of the major hurdles in microbiome science. Before this cross-kingdom communication can be used to treat neurological illnesses, it is important to better understand the intricacy of the microbiota and its biochemical interaction with the host. This intricacy may be reflected in the conflicting results found in various investigations. The complex interplay exists between the metabolic pathways as well as bacterial and human metabolism. For instance, the abundance of SCFAs and bile acids in the gut is intrinsically linked to the production of intestinal neurotransmitters, and inflammatory molecules like nitrate encourage the metabolism of choline by choline-utilizing bacteria. This evidence suggests that the psychotropic effect of a particular metabolite may be tightly dependent on the presence of other metabolites. It is therefore difficult to determine how much of the observed impact on depressive behavior can be attributed to gut microbial metabolism. De Cremoux emphasized in a recent study from Seventure that it’s critical to remember that some microorganisms in the human microbiome are found in lesser numbers. The essential participants in a given condition could be concealed among less frequent species, despite the fact that it is easier to gather data on the most prevalent strains. Studies on the microbiome generate enormous volumes of data that are highly variable. Numerous studies in this area result in contradicting findings due to this variability.

Similarly, there are still significant gaps in our knowledge of how epigenetic mechanisms contribute to depression. The majority of studies describing epigenetic alterations in patients with depression have concentrated on DNA methylation and less research has been conducted on histone modifications and non-coding RNAs. The majority of research has used peripheral tissue, while those that have used post-mortem brain have typically used brain homogenate and smaller cohort size. These factors have made it challenging to confirm depression-related epigenetic changes across cohorts. The challenge has only been exacerbated by additional factors that are already part and parcel of psychiatric research, such as the polygenic architecture of depression, retrospective recollection of environmental exposures and symptom-based diagnoses.

Understanding of the pathological mechanisms of depression will advance with the closure of knowledge gaps on the multifactorial interaction between epigenetics, gut microbiome and their antidepressant effects, which may also help in the development of more sophisticated pharmacological approaches. Future study is critical to fully comprehend the gut flora, as shown by the aforementioned discoveries and the recent spike in media interest in gut health. Effective and widely available therapies for anxiety and depression would be helpful to millions of individuals worldwide since these conditions are becoming more prevalent globally.

## Conclusion

The gut-brain axis is a dynamic, complex, and bidirectional communication network between the gut and brain. Since gut microbiota is a crucial regulator of the gut-brain axis, it is often referred to as microbiota-gut-brain axis. Some metabolites produced by the microbiome (SCFA, bile acids, tryptophan metabolites, vitamins, etc.) can cross BBB and exert a direct and indirect effect on the brain. Various preclinical and clinical studies revealed gut dysbiosis as one of the etiopathological factors in numerous metabolic, psychiatric, and neurodegenerative diseases. Gut metabolites can regulate the host genome by modifying host epigenome through various epigenetic mechanisms such as DNA methylation or acetylation, histone modification, RNA associated silencing, etc. butyrate, one of the SCFAs, is a well-known HDAC inhibitor, choline acts as methyl donor for DNA methylation, amino acids produced by gut bacteria acts as a precursor for the synthesis of neurotransmitters such as GABA, glutamate, epinephrine, nor-epinephrine, and dopamine. Depression is a multifactorial psychiatric disorder, and a plethora of studies has proved the separate involvement of gut microbiota as well as epigenetics as a key player in its etiopathogenesis. Despite the fact that gut microbiome is essential for modulating the host’s epigenetic processes, much research is required to understand the underlying molecular mechanisms and their biological effects on the host. For example, which bacterial species exhibit symbiotic relationships, how they interact with one another, and how their metabolites contribute to epigenetics as well as depression? Therefore, further research on particular gut microbial metabolites that can alters the host epigenome will provide new insight into human health and diseases. Additionally, studies examining the role of gut microbiome in depression and its impact on health conditions are becoming more prevalent. This may pave the way for the discovery of novel therapeutic mechanisms that could aid in the treatment and prevent depression by restoring the altered intestinal microbiome to a healthy state.

## Author contributions

NB, AM, and KT drafted the manuscript. NB revised the manuscript critically for important intellectual content and made figures. SS, MS, SBS, and DK has made significant contributions to the development of the article’s concept and design, as well as to its peer review and editing. All authors read and approved the final manuscript.
